# Promoting spinal cord injury repair by using ZnO@MOFs nanozymes functionalized hydrogel through the ROS microenvironment regulating pathway

**DOI:** 10.1093/rb/rbaf095

**Published:** 2025-09-13

**Authors:** Jiaxin Ding, Binbin Gao, Zelin Sang, Zhen Dai, Zhenhua Chen, Xifan Mei

**Affiliations:** Dalian Medical University, Dalian, 116044, China; Third Affiliated Hospital of Jinzhou Medical University, Jinzhou, 121000, China; Liaoning Provincial Key Laboratory of Medical Tissue Engineering, Jinzhou Medical University, Jinzhou, 121001, China; Jinzhou Central Hospital, Jinzhou, 121000, China; Liaoning Provincial Key Laboratory of Medical Tissue Engineering, Jinzhou Medical University, Jinzhou, 121001, China; Jinzhou Medical University, Jinzhou, 121001, China; The First Affiliated Hospital of Jinzhou Medical University, 121001, China; Liaoning Provincial Key Laboratory of Medical Tissue Engineering, Jinzhou Medical University, Jinzhou, 121001, China; Jinzhou Medical University, Jinzhou, 121001, China; Dalian Medical University, Dalian, 116044, China; Third Affiliated Hospital of Jinzhou Medical University, Jinzhou, 121000, China; Liaoning Provincial Key Laboratory of Medical Tissue Engineering, Jinzhou Medical University, Jinzhou, 121001, China

**Keywords:** oxidative stress, ferroptosis, near-infrared, nanozymes, spinal cord injury

## Abstract

Spinal cord injury (SCI) is a kind of health problem characterized by oxidative stress and neuronal apoptosis, which pose major challenges to the recovery of patients. Recently, the application of photothermal nanotechnology in medicine has opened up exciting new avenues for the treatment of SCI. This innovative approach leverages the unique properties of nanomaterials to enhance therapeutic outcomes. In our study, we developed a novel nanotherapeutic system named ZnO-ZIF8@H, which is designed to deliver targeted neuroprotective effects. We meticulously evaluated its performance under near-infrared (NIR) irradiation, which is known to promote local heating and stimulate biological processes. The data indicated that the application of ZnO-ZIF8@H combined with NIR irradiation significantly reduced oxidative stress levels in the affected tissues. This was evidenced by a marked decrease in malondialdehyde (MDA) levels, a well-known indicator of lipid peroxidation and cellular damage. Simultaneously, the treatment notably enhanced the activity of superoxide dismutase (SOD) and glutathione (GSH) enzymes. These findings suggest that ZnO-ZIF8@H+NIR could both protect cells from oxidative damage and boost the internal antioxidant defenses, highlighting its potential as an effective therapeutic strategy for mitigating secondary injuries following spinal cord trauma. It also suppressed neuronal apoptosis, as evidenced by TUNEL staining and decreased Cleaved-Caspase3 expression in NeuN-positive neurons. These results indicated that ZnO-ZIF8@H+NIR effectively reduces secondary damage from SCI by alleviating apoptosis and oxidative stress, offering a promising approach for the therapy of SCI.

## Graphical abstract

**Figure:**
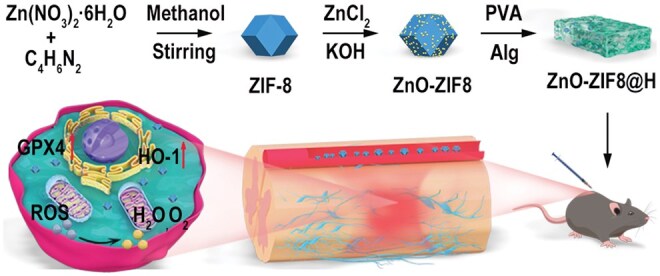

## Introduction

Because of the lack of effective treatments currently, spinal cord injury (SCI) is an extremely debilitating health problem which significantly impairs patients’ physiological functions and quality of life [1–3]. The pathological process of SCI encompasses intricate secondary impairment mechanisms, such as inflammatory reaction, oxidative stress, cell apoptosis and hematomas which are largely driven by mitochondrial dysfunction [4–6]. Therefore, promoting the restoration of mitochondrial function has become a critical focus in the development of SCI treatments [7–9]. Injectable hydrogels have attracted extensive attention in the biomedical field due to their excellent performance [10, 11]. The ideal injectable hydrogel for spinal cord injury repair should have good mechanical properties and biocompatibility [12]. Sodium alginate (Alg), as a natural polysaccharide, has biocompatibility, ion crosslinking ability and loading function. Polyvinyl alcohol (PVA), as a synthetic polymer, can improve mechanical properties and chemical stability. Through physical cross-linking, they have constructed a kind of hydrogel with biological activity and engineering characteristics, which shows potential in neural tissue engineering. Although injectable PVA-Alg hydrogel has been used in the repair of SCI, the current research focus is to further enhance its neuroprotective and antioxidant properties through multifunctional nano enzymes. In recent years, metal-organic frameworks (MOFs), known for their high surface area, controllable drug loading capabilities and excellent photothermal conversion efficiency, have shown great potential in biomedical applications [13–15]. However, how to integrate the functional properties of MOFs into the specific therapeutic needs of SCI remains an important research challenge.

Among different kinds of MOFs, zinc-related MOFs are attractive for the capacity to release Zn^2+^ in a regulated manner. Taking ZIF-8 as an example, its core components (Zn^2+^ and 2-methylimidazole) exhibit excellent biocompatibility in physiological environments. In addition, based on the pathological characteristics of SCI, the acidic microenvironment formed by ischemia and hypoxia in the injured area can trigger the structural dissociation of ZIF-8, allowing it to release loaded drugs as needed, thereby achieving precise targeted drug delivery to the injured area, significantly reducing systemic toxicity while enhancing therapeutic efficacy. Zn^2+^ is important metal ions which are critical to cellular signaling, oxidative stress resistance and mitochondrial protection. Studies have shown that an appropriate amount of Zn^2+^ can effectively alleviate inflammation and oxidative damage in SCI [16–19]. At the same time, zinc ions are significant in suppressing ferroptosis after SCI. However, achieving precise and controlled release of zinc ions *in vivo* remains a challenge. Near-infrared (NIR) light, with its excellent tissue penetration and low toxicity, are attracting in light-responsive drug delivery systems. Therefore, designing an NIR-responsive ZnO@MOFs nanozymes functionalize hydrogel system for SCI treatment holds significant potential value.

**Figure 1.**
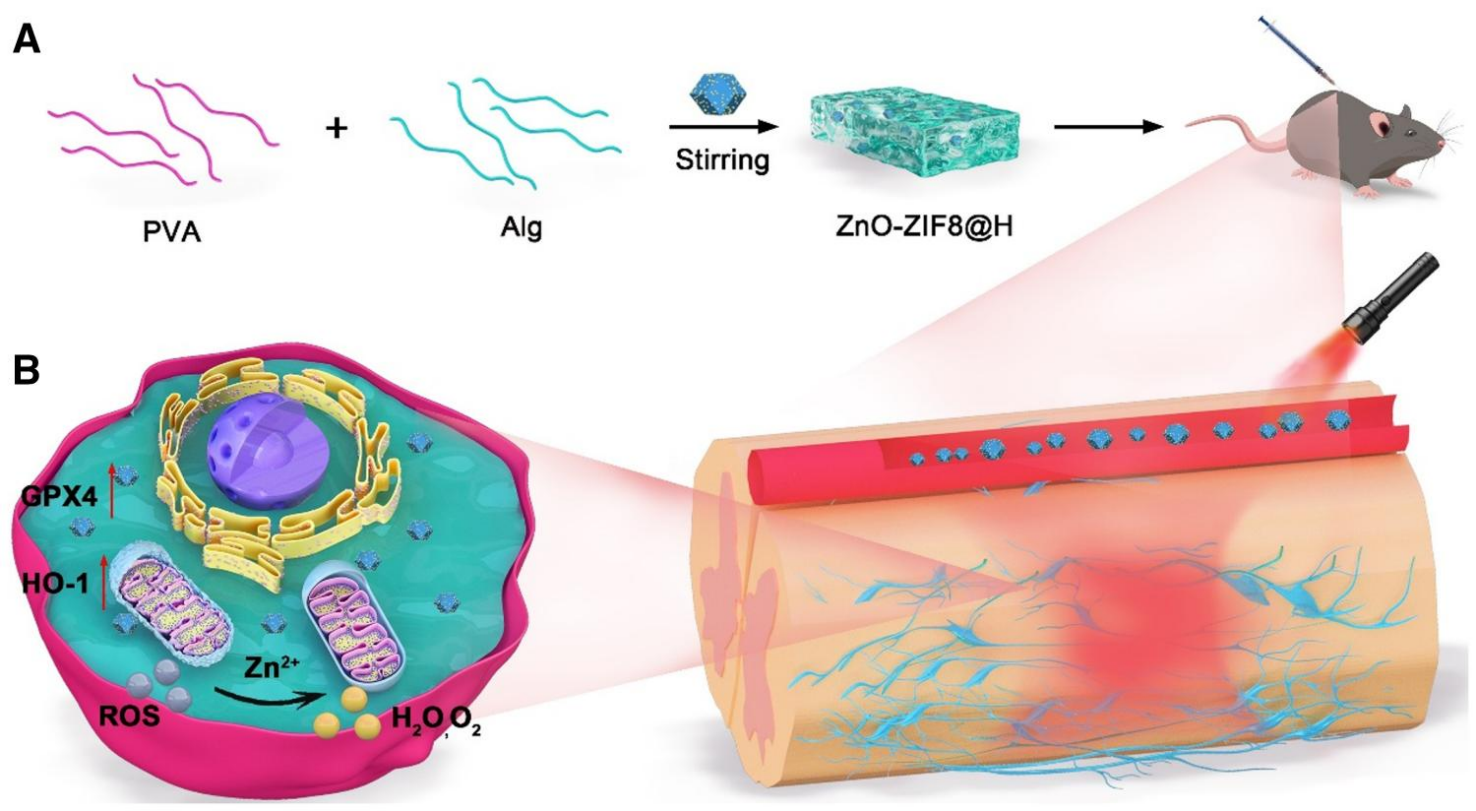
(**A**) The schematic preparation steps of ZnO-ZIF8@H. (**B**) The release of Zn^2+^ from ZnO-ZIF8@H to the injury, and effectively remove ROS in neurons.

Thus, to resolve these problems, this study proposes a NIR-responsive ZnO-MOF nanoplatform for the treatment of SCI. By leveraging NIR light activation, this system will enable precise and controllable Zn^2+^ release while specifically targeting mitochondrial functions to mitigate cellular damage and apoptosis. The prepared steps and biological function of ZnO-MOF@H in SCI treatment was showed in Figure 1A. ZnO-MOF@H under NIR irradiation and gel swelling can release zinc ions rapidly enriched to the site of injury, which effectively modulates the ROS microenvironment in mitochondria. This further reduced neuronal ferroptosis and promoted nerve regeneration (Figure 1B).

## Materials and methods

### Materials

Zn(NO_3_)_2_·6H_2_O, ZnCl_2_, H_2_O_2_ (30%), PVA, Alg, Zincon and C_4_H_6_N_2_ (2-methylimidazole) were sourced from Aladdin reagent (China). DMEM along with FBS were acquired from Gibco (USA).

### Preparation steps of ZIF–8

Firstly, methanol was used to dissolve 0.803 M C_4_H_6_N_2_ and 0.101 M Zn(NO_3_)_2_·6H_2_O, separately. Next, 2 mL C_4_H_6_N_2_ solution was dropped into 2 mL Zn(NO_3_)_2_·6H_2_O solution. Then, stirred the obtained mixture at 200 r/min for 1 h at ambient temperature. Finally, the products were centrifuged (13 000 r/min) and washed by methanol for 2∼3 times. To get ZIF-8 powders, the collected products were freeze-dried.

### Preparation steps of ZnO-ZIF8 and ZnO-ZIF8@H

First, ZIF-8 (20 mg) was dispersed in 1 mL of methanol under sonication. Meanwhile, ZnCl_2_ (10 mg) was dispersed in 1 mL methanol. Afterwards, mix ZnCl_2_ and ZIF-8 with potassium hydroxide and stir continuously for 0.5 h. Next, the product was collected by centrifugation (13 000 r/min, 5 min). After washing, ZnO-ZIF8 was yielded. For the preparation of the hydrogel, dissolved 800 mg PVA in 10 mL distilled water. Then, put the PVA solution at 90°C for 1 h. After that, Alg (0.15 g) was added into PVA solution at 70°C. Then, put ZnO-ZIF8 into the PVA-Alg mixture under stirring for 1 h. After gelation, ZnO-ZIF8@H was obtained.

### Characterization of the samples

Dynamic laser scattering (DLS) was performed by a Malvern (UK) Nano ZS90 instrument to measure the sample size. Sample’s composition was confirmed by fourier transform infrared (FTIR) spectroscopy using a Gangdong spectrometer (FTIR-650S, China). The Lambda 605S ultraviolet-visible (UV–Vis) spectrophotometer (PerkinElmer) was used to record peak position of the samples. Scanning electron microscope (SEM, S4800, Hitachi, Japan) was used to characterize the morphology of the samples. The morphological features of the synthesized materials were characterized via transmission electron microscopy (TEM, JEM-1200EX, JEOL, Tokyo, Japan). The crystalline properties of the samples were tested by X-ray diffraction (XRD).

### The release of ZnO *in vitro*

ZnO-ZIF8 was dispersed in a PBS solution. Subsequently, the mixture was transferred into a dialysis bag. Then, it was submerged in 50 mL PBS solution in a tube. Such tube was put in a shaker with water-bath (100 r/min). For testing the release of ZnO, samples (4 mL each time) were drawn from the tube with or without NIR irradiation, and further analyzed by using UV–Vis.

### Animals

The experiment involved C57BL/6J mice, equally male and female, weighing 25–30 g and 6–8 weeks old. Mice were purchased from and housed at our university’s animal center. All procedures received approval (Approval No. SYXK 2019-0007). 1% sodium pentobarbital (dosage of 50 mg/kg) was used for anesthesia through the intraperitoneal injection. After the dorsal fur was shaved, the skin was disinfected with povidone-iodine. A laminectomy (at the T9/T10 vertebral level) was carried out to expose the thoracic spinal cord, creating a moderate contusion model using a 50 Kdyne impactor. At the end of the surgery, wounds were sutured and disinfected again with povidone-iodine, followed by postoperative care. Randomized grouping and double-blind testing ensured unbiased results. Mice were randomly assigned to four groups: Sham, the SCI group, the ZnO-ZIF8@H group and the ZnO-ZIF8@H + NIR group. The treatment group was injected with 3 μL of ZnO-ZIF8@H at the site of spinal cord injury, the SCI group was injected with an equal amount of PBS, and the Sham group was not injected. The ZnO-ZIF8@H + NIR group was further subjected to a NIR irradiation regimen: irradiation for 5 min three times per day with 5 min intervals between consecutive irradiations.

### Cell cultivation

PC12 cells were cultivated at 37°C in an environment with 5% CO_2_. The culture medium was a high glucose DMEM, which was fortified with 1% penicillin-streptomycin and 10% FBS. H_2_O_2_ was used to induce oxidative stress. The cells were partitioned into 4 groups: Control, ZnO-ZIF8, ZnO-ZIF8@H, ZnO-ZIF8@H + NIR, exposed to 100 μmol/L H_2_O_2_. This experimental setup was devised to assess the combined impacts of these agents in alleviating cell damage caused by oxidative stress.

### Cell staining with calcein AM/PI

After the PC12 cells were co-cultured with the materials for 1, 3 and 5 days, PC12 cells’ viability was evaluated by means of the Cytotoxicity Assay Kit (Calcein AM/PI Beyotime). Incubated the cells together with the working solution for 0.5 h. Then, analysis the samples using a fluorescence microscope.

### Cell counting kit-8

96-well plates were used to culture PC12 cells at a density of 5 × 10^4^ mL^−1^. Cells were cultivated in the co-presence with ZnO-ZIF8@H and ZnO-ZIF8@H + NIR. After being incubated for one day, 10 µl CCK-8 was introduced into each plate well. Cell Counting Kit-8 was purchased from Beyotime, China (C0038). The incubator was then maintained at 37°C for a further 2 h. Subsequently, the absorbance (at 450 nm) of the solution in each well was recorded.

### Cell immunofluorescence staining

For PC12 cells in all groups, 4% paraformaldehyde was used to fix them for 15 min. Then, they were washed by PBS. Next, 0.1% Triton X-100 was applied to permeabilize the cells over a 10 min interval, and they were blocked with 5% BSA for 0.5 h. Then, incubated the cells with primary antibodies-anti-GPX4 (1:200, DF6701, Affinity) and anti-HO-1 (1:500, BF8020, Affinity) at 4°C overnight. After washing with PBS, secondary antibodies were applied for 2 h, followed by DAPI staining for nuclear visualization. Confocal laser scanning microscope (CLSM) was used to record the cell images. Subsequently, oxidative stress levels in each group were assessed.

### Immunofluorescence staining

For spinal cord cryosections, 0.3% Triton X-100 was used for a 10 min treatment, and then, they were blocked with 2% BSA for 2 h. Sections were left to incubate at 4°C throughout the night with primary antibodies, namely anti-Cleaved-Caspase3 (1:200, BF0711, Affinity), anti-NF200 (1:500, 18934-1-AP, Proteintech), anti-NeuN (1:500, DF6145, Affinity) and anti-GFAP (1:500, 60190-1-1 g, Proteintech). Subsequently, secondary antibodies were applied for 2 h, and then, DAPI staining was done for 15 min. A Leica upright fluorescence microscope was used for imaging.

### SOD, MDA, GSH assay

To evaluate superoxide dismutase (SOD) activity, levels of malondialdehyde (MDA) and the content of reduced glutathione (GSH), we carried out the Kit Assay (BC0170 for SOD, BC0020 for MDA and BC1157 for Reduced GSH; all from Solarbio, China). Each assay was performed in strict compliance with the manufacturer’s guidelines.

### Basso mouse scale test

The hindlimb motor performance of mice was measured both before the operation and on 1, 3, 7, 14, 21 and 28 days after SCI. This assessment was carried out by employing the BMS (Basso Mouse Scale) scoring system. The mice were put in open surroundings. Three independent researchers, who were unaware of which treatment groups the mice belonged to, then evaluated the mice’s performance. A double-blind protocol was strictly followed throughout the study.

### Δ*ψ*M assay

The samples were assessed by means of the JC-1 Δ*ψ*M Detection Kit (J604, AAT Bioquest, Suzhou). Treated the cells with a staining solution containing JC-1 (2.0 μg/mL) and then incubated at 37°C for 20 min. After washing, fluorescence was analyzed under a Leica DMi8 fluorescence microscope. For normal cells, the aggregated JC-1 in mitochondria presents red fluorescence. In contrast, JC-1 monomers resulted from the decreased membrane potential present green fluorescence. Apoptotic and necrotic cells exhibit lower red/green fluorescence ratios. For each group, a blinded researcher captured five random images, and fluorescence intensity was quantified using ImageJ after consistent threshold adjustment.

### ROS assay

Reactive Oxygen Species (ROS) in PC12 cells were quantified by using DCFH-DA (2′,7′-dichlorodihydrofluorescein diacetate, Elabsciences, Shanghai, China). Cells were incubated at 37°C for 0.5 h in the presence of 50 μM DCFH-DA. After the incubation, collected the cells by centrifugation and rinsed by PBS thoroughly. Then, determined the fluorescence intensity of cells by BioTek plate reader (Synergy LX, USA) under 500 nm excitation wavelength and 525 nm emitting wavelength.

### Measurement of ATP content

ATP Assay Kit (S0027, Beyotime Biotechnology) was used to measure ATP levels. Tissue and cell samples were processed as instructed. ATP detection working solution was added to each well of the opaque 96-well plate (100 μL). After that, to eliminate endogenous ATP, the plate was incubated at ambient conditions for 5 min. Subsequently, 20 μL sample or standard was added. Versa Max microplate reader (Molecular Devices, USA) was used to record the fluorescence intensity (in Relative Light Units, RLU). After that, the recorded fluorescence intensity was used to calculate the ATP content.

### Footprint test

The assessment of motor coordination and gait recovery was evaluated through a footprint test. At the 28th day after spinal cord injury, the mice were gently guided to traverse a straight path on a sheet of white paper. This process was repeated three times. Prior to walking, stained the front paws black, while dyed the hind paws red. As the mice walked along the designated path, their footprints were imprinted on the paper. These footprints were then carefully documented by capturing images with a camera. Once the images were obtained, they were digitized, facilitating a comprehensive and accurate analysis of the gait and motor coordination parameters. This meticulous approach enabled a detailed evaluation of the mice’s recovery progress in terms of their ability to walk and coordinate their movements.

### Statistics

The experiment included three biological repetitions to ensure reproducibility. For statistical analysis between two groups, Unpaired *t*-tests were used. For multiple group comparisons, one-way ANOVA was used. Analyses were performed using GraphPad software8.0.1. Significant differences (compared with Ctrl) were identified for each figure: *P < 0.05, **P < 0.01, ***P < 0.001.

## Results and discussions

### Characterization of the obtained material samples

**Figure 2.**
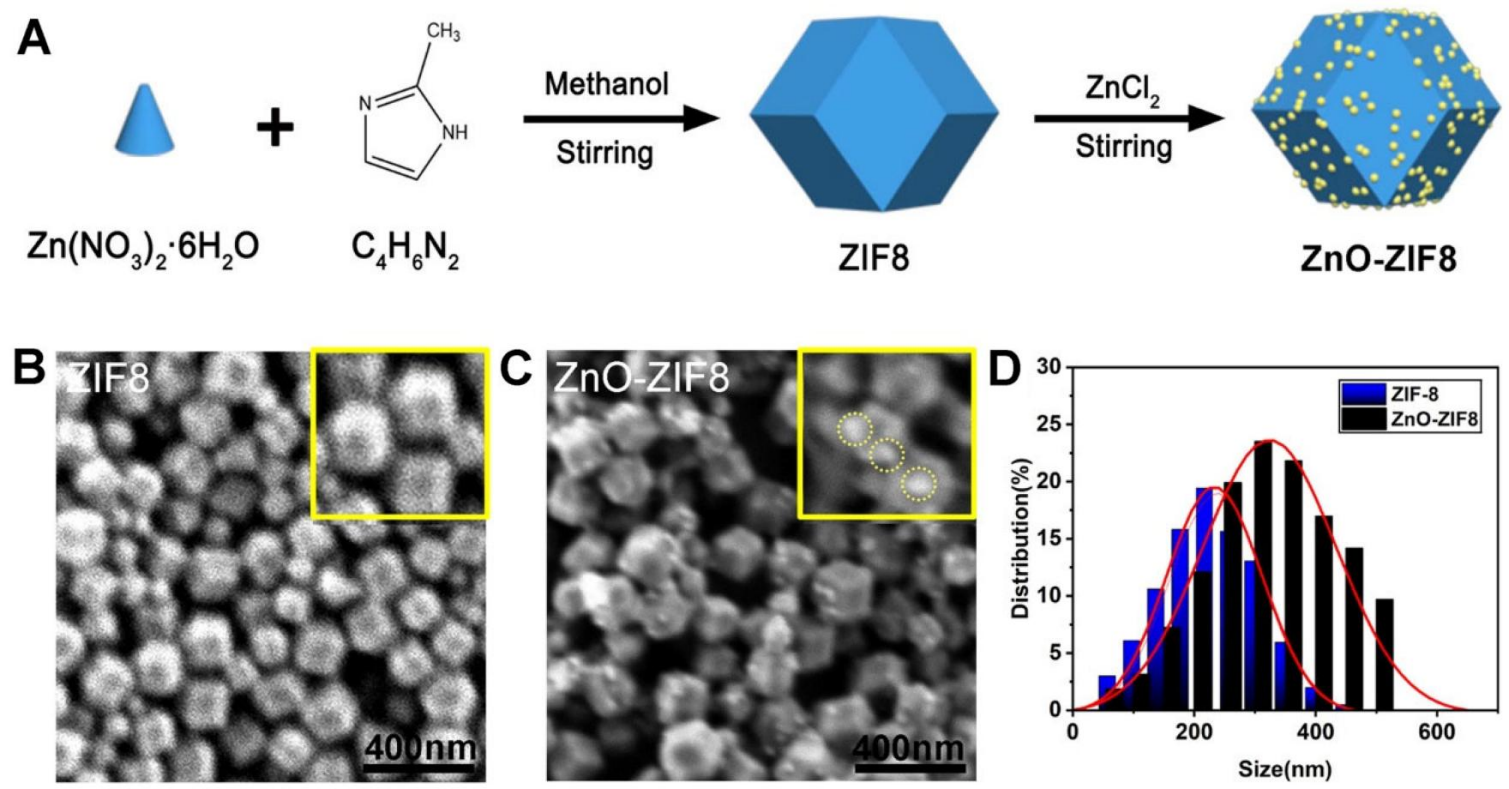
(**A**) Schematic process for preparing ZnO-ZIF8. (**B**) SEM image and the enlarged view (inset) of ZIF8. (**C**) SEM image and the enlarged view (inset) of ZnO-ZIF8. (**D**) DLS results.

ZnO@ZIF8 nanozymes could be prepared by *in situ* reduction technique as illustrated in Figure 2A. To investigate the structure and morphology of ZIF-8 and ZnO@ZIF8 nanozymes, SEM analyses were performed. Figure 2B indicated the obtained ZIF-8 were rhombic dodecahedral with an average size of ∼200 nm. SEM images in Figure 2C confirmed that ZnO nanoparticles had been successfully loaded on the ZnO-ZIF8 nanozymes. Under the characterization of TEM, ZnO-ZIF8 nanozymes presents unique micro-morphological features (Supplementary Figure S1A). The TEM images showed that the ZIF-8 matrix maintains a typical rhombic dodecahedral structure, with well-defined edges and smooth surface. The ZnO nanoparticles are uniformly distributed on the surface of the ZIF-8, and they are spherical, forming close contact with the ZIF-8 surface (Supplementary Figure S1B). The morphology and elemental distribution of nanoparticles were further observed by energy-dispersive spectroscopy (EDS) elemental mapping. Dark-field scanning transmission electron microscopy and energy spectroscopy verified the successful synthesis of ZnO-ZIF8, and the images demonstrated the ZnO nanoparticles grown on the ZIF-8 (Supplementary Figure S1C). Subsequent EDS analysis confirmed the successful construction of ZIF-8 and the introduction of ZnO nanoparticles (Supplementary Figure S1D). DLS indicated that the average size of ZIF-8 was 215 nm, while that for ZnO-ZIF8 nanozymes was 265 nm (Figure 2D). This data further confirmed the decoration of ZnO on ZnO-ZIF8. Supplementary Figure S2 shows a schematic diagram of hydrogel preparation by forming a hydrogen bond network in the PVA-Alg matrix. Schematic diagram of intermolecular forces in pure hydrogel and ZnO-ZIF8@H gel networks (Supplementary Figure S3) indicates that the ZnO-ZIF8@H network includes hydrogen bonds (between PVA and Alg molecules) and chelation interactions between carboxyl groups (from Alg) and metal ions (from ZnO-ZIF8). We demonstrated the internal microstructure of the hydrogel composite system by SEM imaging of the hydrogel, with Supplementary Figure S4 showing the microstructure and elemental distribution of ZnO-ZIF8@H. The SEM images in Supplementary Figure S4A and B reveal the three-dimensional porous framework of the hydrogel, with uniform and continuous pore walls. Pore statistics show an average pore size of 9.5 ± 2.6 µm (Supplementary Figure S5) and a total porosity of 64.9% (Supplementary Figure S4C and D). Elemental mapping of the same region indicates that strong signals for Zn and N are concentrated in the ZnO-ZIF8 nanoparticle regions, confirming that the particles are uniformly embedded in the hydrogel network.

**Figure 3.**
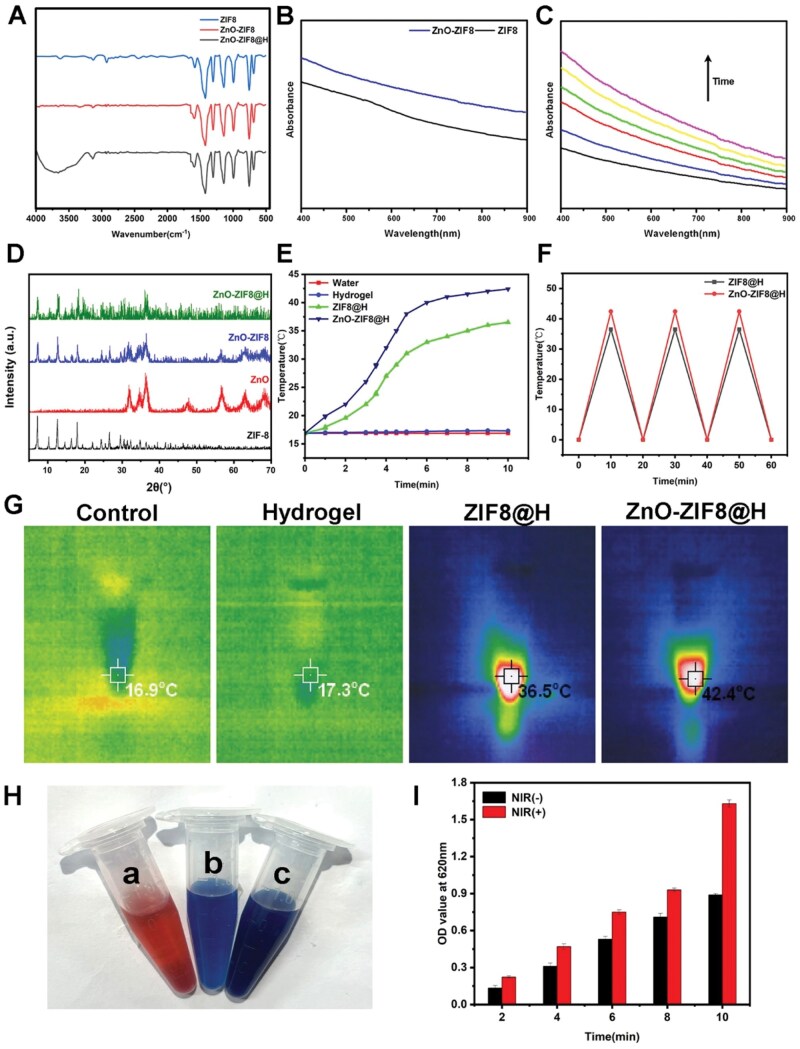
(**A**) FTIR Spectra of ZIF-8, ZnO-ZIF8 and ZnO-ZIF8@H. (**B**) UV–Vis spectra of ZnO-ZIF8 and ZIF-8. (**C**) The released ZnO’s UV–Vis spectra at various time points. (**D**) XRD patterns of different samples. (**E**) Photothermal heating curves of different samples (**F**) Photothermal stability of the samples. (**G**) The images of the various samples with NIR irradiation (808 nm) for 5 min. (**H**) Test of the released Zn^2+^ by color change reaction (a: pure zincon; b: Zincon + Zn^2+^ NIR-; c: Zincon + Zn^2+^ NIR+). (**I**) Absorbance of Zn^2+^ released from ZnO-ZIF8 reacting with zincon at various time points.

The successful synthesis of ZnO-ZIF8@H was further confirmed via FT-IR (Figure 3A). The results of UV–Vis spectra (Figure 3B) indicated that ZnO-ZIF8 presented a broad absorption peak. Subsequently, retardation experiments, as indicated by UV–Vis, verified that the samples could maintain the release of ZnO within the micro-surroundings of spinal cord injury (Figure 3C). XRD analyses of ZnO-ZIF8@H revealed the presence of the ZnO-ZIF8 diffraction peaks, which indicated that the composites retained the intact crystal structures of ZnO and ZIF8 (Figure 3D). ZnO-ZIF8 nanozymes are a nanomaterial with significant photothermal conversion performance, and its unique MOF structure endows it with efficient NIR light response capability [20–23]. Under NIR irradiation, ZnO-ZIF8 can rapidly convert light energy into heat, exhibiting excellent photothermal conversion efficiency. In addition, based on the photothermal curve (Supplementary Figure S6A) and the time constant of system heat transfer ($\tau_{s}$=81.94 s; Supplementary Figure S6B), The photothermal conversion efficiency of ZnO-ZIF8@H was calculated to be 46.62% (refer to supporting information for detailed calculations). Therefore, ZnO-ZIF8@H has excellent photothermal conversion efficiency, making it suitable for the treatment of SCI. To confirm the photothermal effect of ZnO-ZIF8@H, we performed the following experiments: the samples underwent irradiation by a NIR laser (808 nm wavelength, 2.5 W/cm^2^ power density) for 5 min. Thermal imaging camera was used to record the temperature changes of various samples after NIR irradiation (808 nm) for 5 min. Figure 3E and G indicated that compared with 27.2°C for Ctrl and 28.8°C for pure hydrogel, ZIF8@H reached 36.5°C while ZnO-ZIF8@H reached the highest temperature of 42.4°C. Photothermal stability tests were performed three cycles of irradiation experiments on ZnO-ZIF8@H under NIR irradiation (Figure 3F). In each cycle, we observed only minor temperature fluctuations between adjacent peaks, which indicates the stable photothermal conversion efficiency of ZnO-ZIF8@H. Zn^2+^ could be generated caused by the dissociation of ZnO and ZIF-8 with NIR irradiation. Given that zincon can undergo a chromogenic reaction (forming a blue complex) with Zn^2+^, it can be utilized to confirm the generation of Zn^2+^. Figure 3H demonstrated the shift of the zincon solution from red to blue because of Zn^2+^, verifying the successful release of Zn^2+^. Since the blue solution has the characteristic absorption at 620 nm, we systematically compared the absorbance changes of ZnO-ZIF8 nanozymes at different time points under NIR irradiation (NIR+) versus no NIR light (NIR-) conditions. Figure 3I clearly showed the absorbance values of the ZnO-ZIF8 solution under NIR+ were always higher than those under NIR-. This phenomenon indicated that ZnO-ZIF8 could generated more Zn^2+^ with NIR irradiation. In addition, the NIR controlled strategy achieved in this study maintains Zn^2+^ levels within the recognized safety window while ensuring therapeutic doses, effectively avoiding neurotoxicity (Supplementary Figure S7). In summary, ZnO-ZIF8@H had good photothermal properties, and the resulting localized heat under NIR laser irradiation led to the gradual decomposition of ZnO-ZIF8 and accelerated the leaching of Zn^2+^ from ZnO-ZIF8@H for the application in SCI.

The mechanical properties of hydrogels were quantitatively characterized by compression and tensile tests (Supplementary Figure S8A and B). The tensile data showed that the maximum breaking force of the ZnO-ZIF8@H was significantly higher than that of the hydrogel, which indicated that ZnO-ZIF8 nanozymes enhanced the ductility of the hydrogel (Supplementary Figure S8A). The compression test data show that the force values of hydrogel and ZnO-ZIF8@H are basically the same in the small displacement interval, while the force value of ZnO-ZIF8@H hydrogel gradually exceeds that of the pure hydrogel with the increase of displacement, which intuitively reflects that it has a stronger load-bearing capacity under the condition of large deformation (Supplementary Figure S8B). The introduction of ZnO-ZIF8 nanozyme optimized the mechanical properties of the hydrogel significantly.

First, we investigated the modulus-frequency relationship and rheological properties of hydrogels and ZnO-ZIF8@H using the frequency scanning method (Supplementary Figure S8C and D). The storage modulus (G′) of both hydrogel and ZnO-ZIF8@H exceeds the loss modulus (G″) over the entire frequency range, which confirms their stable gel state. The rheological properties of PVA solutions, hydrogels and ZnO-ZIF8@H were characterized by assessing the flowability using the tilted bottle test (Supplementary Figure S9). The results showed that the PVA solution has flowability. The prepared hydrogels retained their fluidity; however, the gel residue adhering to the bottom and walls of the bottles indicated a significantly higher viscosity than that of the pure PVA solution. In contrast, the newly synthesized ZnO-ZIF8@H showed a fast-gelling behavior without the need for prolonged aging, as evidenced by the stable, nonflowing state.

Medical hydrogels are expected to exhibit excellent swelling performance, characterized by both appropriate swelling capacity and remarkable stability. Specifically, they should maintain a stable swollen state in physiological fluids, ensuring minimal fluctuations in swelling ratio even under subtle environmental changes (such as small fluctuations in temperature and pH), which is critical for reliable medical applications. To assess this property, the swelling rates of the hydrogels in PBS solution at 4°C and 37°C (Supplementary Figure S10). The results showed swelling rates of 385% at 4°C and 435% at 37°C, demonstrating that the hydrogel not only achieves optimal swelling capacity but also maintains stable performance across temperatures.

### Cytotoxicity of ZnO-ZIF8 and ZnO-ZIF8@H

**Figure 4.**
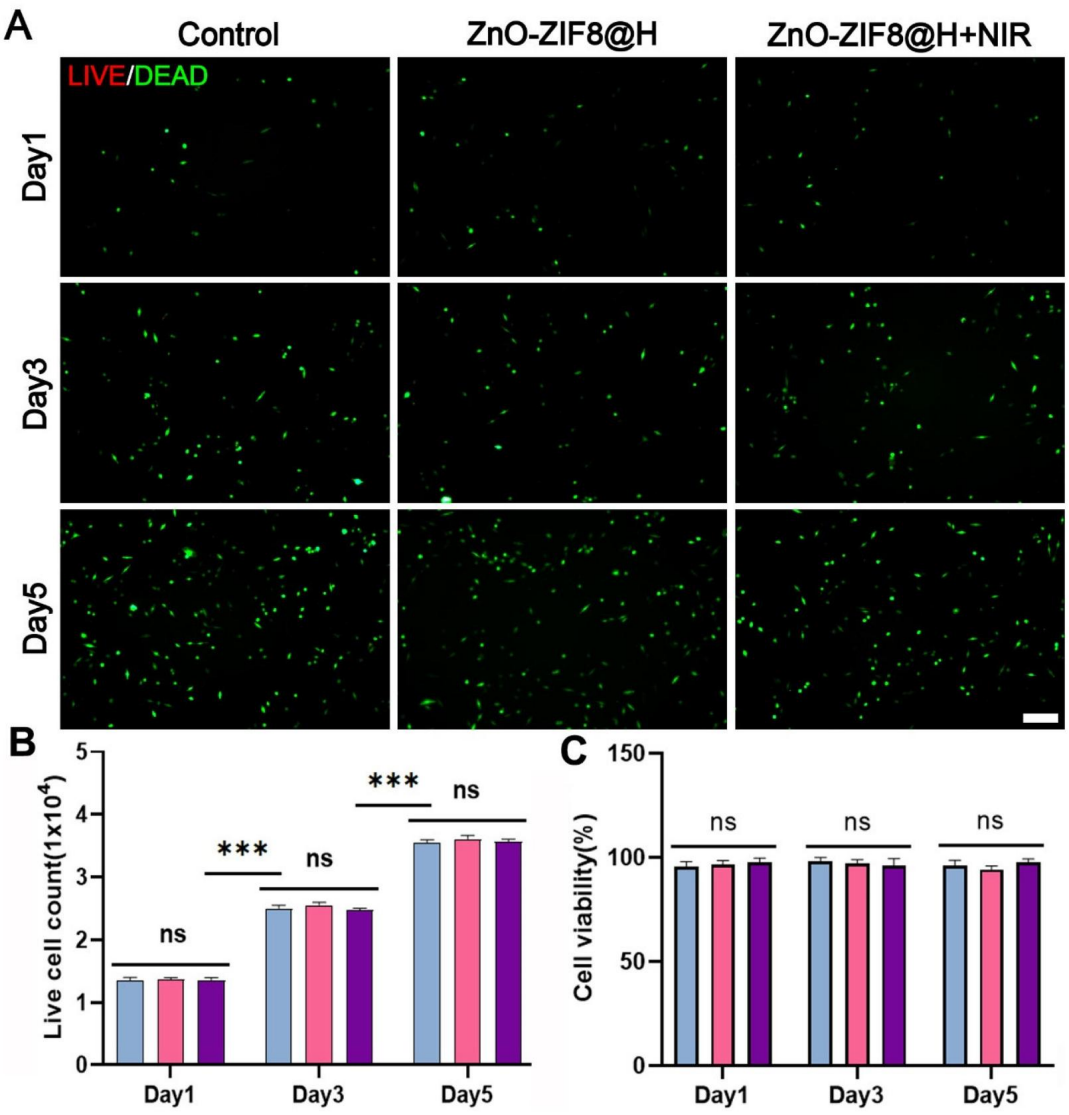
(**A**) Fluorescent images observed via live/dead staining demonstrated the survival of PC12 cells co-cultured with ZnO-ZIF8 and ZnO-ZIF8@H, bar = 200 μm. (**B**) CCK-8 results. (**C**) Cells counting analysis. (*n* = 3, ns represents no significant difference, ***P < 0.001).

ZIF-8 has been reported as multifunctional, biocompatible and stable carrier for active components [24–27]. To evaluate the *in vitro* cytotoxicity of ZnO-ZIF8 and ZnO-ZIF8@H under NIR irradiation, we analyzed PC12 cells using a cytotoxicity detection kit and the CCK-8 method. As shown in Figure 4A, most of the cells maintained a good survival status during the 5-day incubation. Figure 4B indicates no statistically significant variations were detected among the groups. Moreover, the cell density grew as time elapsed. The outcomes of the CCK-8 experiments (Figure 4C) indicated that at different time intervals, no significant disparities in cell proliferation activity were demonstrated by any of the experimental groups. These results indicated that the cell proliferation in all groups remained similar levels at each time points. In summary, ZnO-ZIF8@H exhibited good biosafety properties and provided a suitable micro-surrounding for PC12 cells.

### Mitochondrial function recovery evaluation *in vitro*

**Figure 5.**
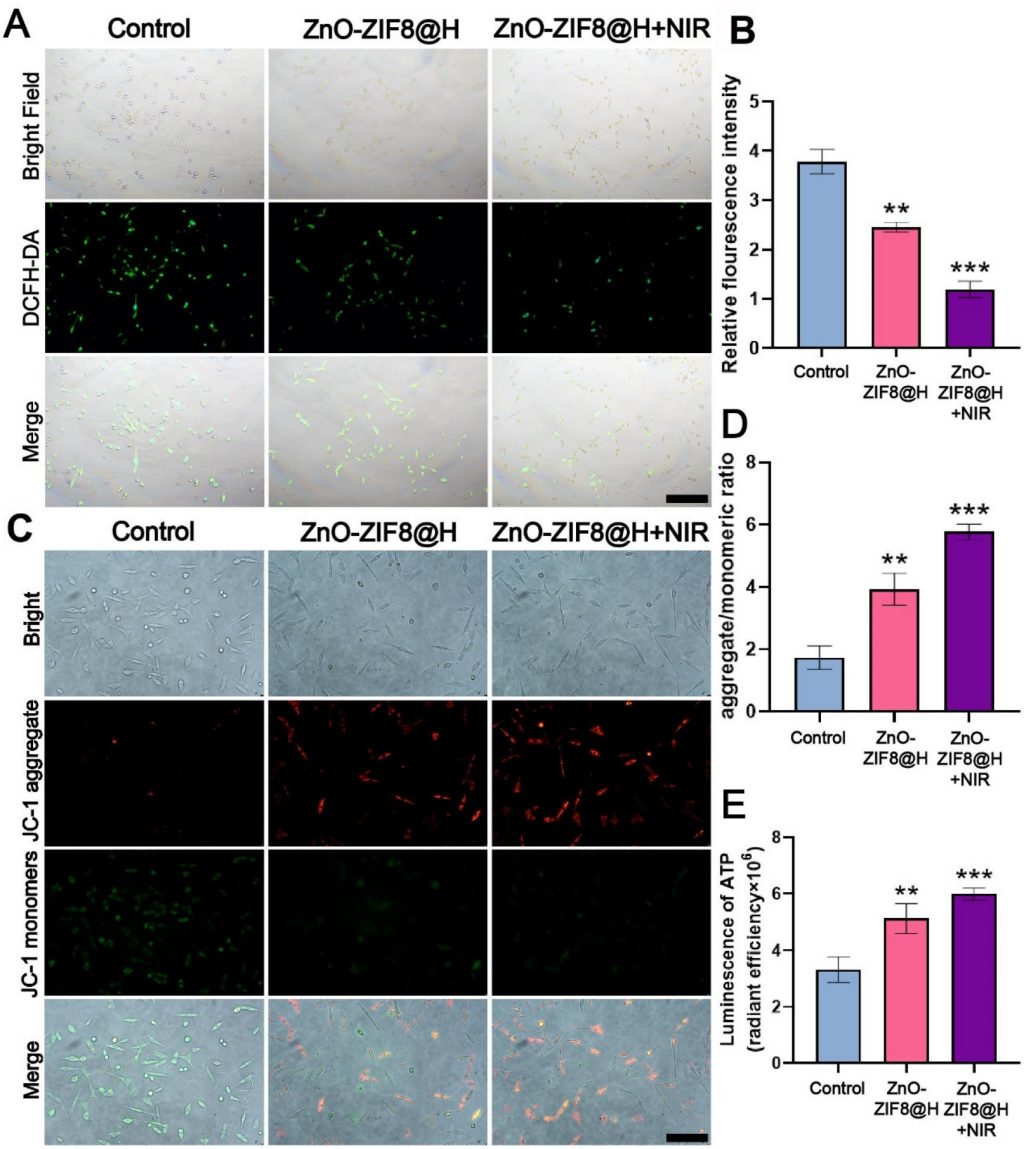
(**A**) DCFH-DA probe detection. (**B**) Analysis of the fluorescence intensity in images in (A). (**C**) ZnO-ZIF8@H repairs mitochondrial dysfunction in PC12 cells under oxidative stress. (**D**) The quantitative assessment of the images in (C). (**E**) ATP production in PC12 cells. Scale bars (in A and C) =100 μm. (*n* = 3, **P < 0.01, ***P < 0.001).

Spinal cord injury (SCI) refers to a severe traumatic disorder of the central nervous system. Notably, oxidative stress plays a crucial role in the advancement of SCI [28–30]. Oxidative stress refers to the damage inflicted on cells and tissues, which results from an imbalance between the generation and elimination of free radicals. This imbalance leads to an overabundance of free radicals, overwhelming the natural antioxidant defense mechanisms. Consequently, these highly reactive molecules can initiate a cascade of harmful reactions, attacking cellular components such as lipids, proteins and DNA, ultimately causing extensive damage at both the cellular and tissue levels [31–33]. Therefore, effective scavenging of reactive oxygen species may become a key measure in the treatment of spinal cord injury. In this experimental work, initially, the DCFH-DA probe was utilized to detect the levels of reactive oxygen species within the nerve cells. As shown in Figure 5A, ROS levels within PC12 cells were significantly increased after H_2_O_2_ treatment, and the addition of ZnO-ZIF8@H or ZnO-ZIF8@H + NIR significantly reduced ROS production (Figure 5B). Impaired function of mitochondria would exacerbate spinal cord injury [34]. In addition, energy depletion of nerve cells impairs their ability to repair and promotes the progression of spinal cord injury. ROS production is important to the impairment of mitochondrial function. Therefore, as showed in Figure 5C, we proceeded to evaluate mitochondrial function through the measurement of mitochondrial membrane potential. With the aid of the fluorescent probe JC-1, we observed mitochondrial membrane potential changes. In normal mitochondria, the aggregation of fluorescent probe caused by membrane potential would make it emit red fluorescence. However, in treated neuronal cells, with the impairment of mitochondrial function, fluorescence of JC-1 probe shifted to green from red, indicating a decrease in membrane potential. In NIR group, the higher red fluorescence intensity than those of the remaining groups might relate to the accelerated release of Zn^2+^ after NIR light irradiation (Figure 5D).

To assess the mitochondrial function more comprehensively, we further used an ATP assay kit to measure the effect of ZnO-ZIF8@H on ATP production in PC12 cells. Figure 5E showed that ZnO-ZIF8@H + NIR effectively promoted ATP production in PC12 cells. These results indicated that ZnO-ZIF8@H + NIR could protect the mitochondrial function of PC12 cells and further enhance ATP’s generation by altering the microsurroundings of oxidative stress.

### ZnO-ZIF8@H ameliorates H_2_O_2_-induced ferroptosis in neuronal cells

Ferroptosis represents a kind of programmed cell death reliant on iron [35–37]. In the context of SCI, abnormal iron metabolism leads to intracellular Fe overload, which produces substantial amounts of ROS via the Fenton reaction. This triggers lipid peroxidation, ultimately resulting in neuronal ferroptosis. Additionally, lipid peroxidation can impair mitochondrial function, further disrupting the functional homeostasis of neurons, thereby promoting the progression of SCI. Therefore, inhibiting ferroptosis could reduce neuronal cell loss following SCI and potentially provide a pathway for spinal cord neurofunctional recovery.

**Figure 6.**
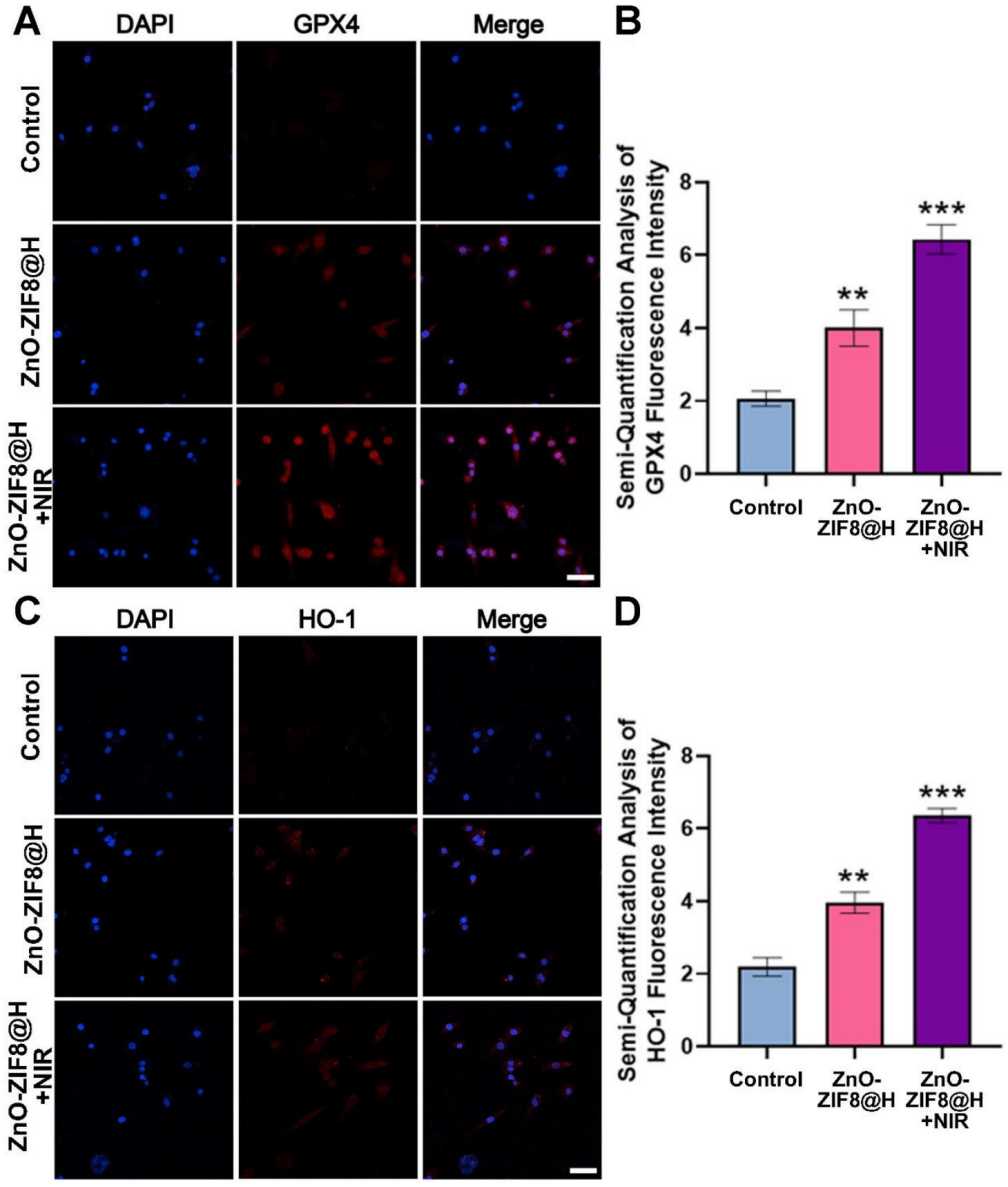
(**A**) CLSM images of GPX4 expression in PC12 cells. (**B**) GPX4 intensity analysis. (**C**) CLSM images of HO-1 expression in PC12 cells. (**D**) HO-1 intensity analysis. The bars are both 50 μm. (*n* = 3, **P < 0.01, ***P < 0.001).

GPX4 and heme HO-1 are closely related to ferroptosis and both play important roles in the treatment of SCI. GPX4 is a pivotal molecular entity in the suppression of ferroptosis, and it is able to reduce lipid peroxidation to nontoxic lipids and alcohols, thus, preventing the propagation of lipid peroxidation, and playing a central protective role in the defense of neuronal cells against ferroptosis. As indicated in Figure 6A and B, the treatment with H_2_O_2_ led to a remarkable reduction of GPX4 within neuronal cells. On the contrary, ZnO-ZIF8@H and ZnO-ZIF8@H + NIR treatments resulted in a significant increase in GPX4 expression level. In addition, GPX4 expression levels were highest in the ZnO-ZIF8@H + NIR group (Figure 6A and B). HO-1 also plays an important regulatory role in the ferroptosis process, which can metabolize hemoglobin to produce carbon monoxide, bilirubin and free iron. Bilirubin is an antioxidant that reduces intracellular oxidative stress, thus, indirectly inhibiting ferroptosis; free iron induces the expression of ferritin, which chelates excess iron, reduces intracellular free iron content, and decreases iron-dependent lipid peroxidation, thus, inhibiting ferroptosis. Subsequently, we evaluated the role of ZnO-ZIF8@H in attenuating H_2_O_2_-induced ferroptosis in neuronal cells by quantitatively analyzing HO-1 expression through immunofluorescence staining (Figure 6C and D). The expression of HO-1 in neuronal cellular entities was upregulated by ZnO-ZIF8@H and ZnO-ZIF8@H + NIR treatments, suggesting that ZnO-ZIF8@H and ZnO-ZIF8@H + NIR could effectively alleviate the pathological process of ferroptosis, and thus, treat spinal cord injury.

### ZnO-ZIF8@H reduces apoptosis and promotes neuronal regeneration after spinal cord injury

**Figure 7.**
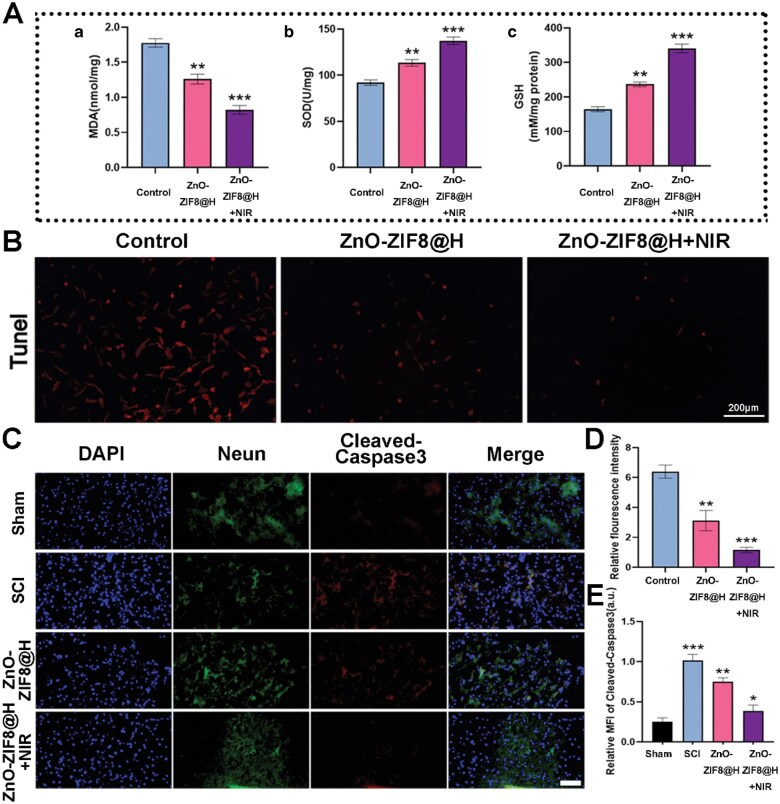
(**A**) Analysis of MDA, SOD and GSH in various groups. (**B**) TUNEL apoptosis results. (**C**) Fluorescence microscopy images of ZnO-ZIF8@H and ZnO-ZIF8@H+NIR treated spinal cord tissue sections, bar = 50 μm. (**D**) Quantification of TUNEL fluorescence intensity. (**E**) Cleaved-Caspase3’s quantification results. (*n* = 3, *P < 0.05, **P < 0.01, ***P < 0.001).

Ferroptosis exerts a vital influence on modulating the oxidative stress state inside cells. We measured the oxidative stress status of each group of cells and the data showed that ZnO-ZIF8@H + NIR markedly reduced the production of MDA, a product of lipid peroxidation, while promoting the activity of the antioxidant enzyme SOD. Additionally, ZnO-ZIF8@H + NIR enhanced the levels of GSH, which is involved in free radical scavenging and various antioxidant reactions (Figure 7A). And with the increase in intracellular oxidative stress, the apoptosis process within the cells is also promoted [38–40]. We performed TUNEL staining and observed that ZnO-ZIF8@H + NIR markedly reduced neuronal apoptosis induced by H_2_O_2_ stimulation (Figure 7B and D). We also examined the expression of the apoptosis protein Cleaved-Caspase3 in neurons marked by NeuN in different groups of mice using immunofluorescence analysis. The expression of Cleaved-Caspase3 was significantly elevated in the mice with SCI, whereas ZnO-ZIF8@H + NIR treatment markedly reduced Cleaved-Caspase3 expression in injured mice. This indicates that ZnO-ZIF8@H + NIR exhibits excellent anti-neuronal apoptosis capabilities (Figure 7C and E). Subsequently, we evaluated the ability of ZnO-ZIF8@H to promote neuronal regeneration. The morphological changes among the groups exhibited significant biological differences.

**Figure 8.**
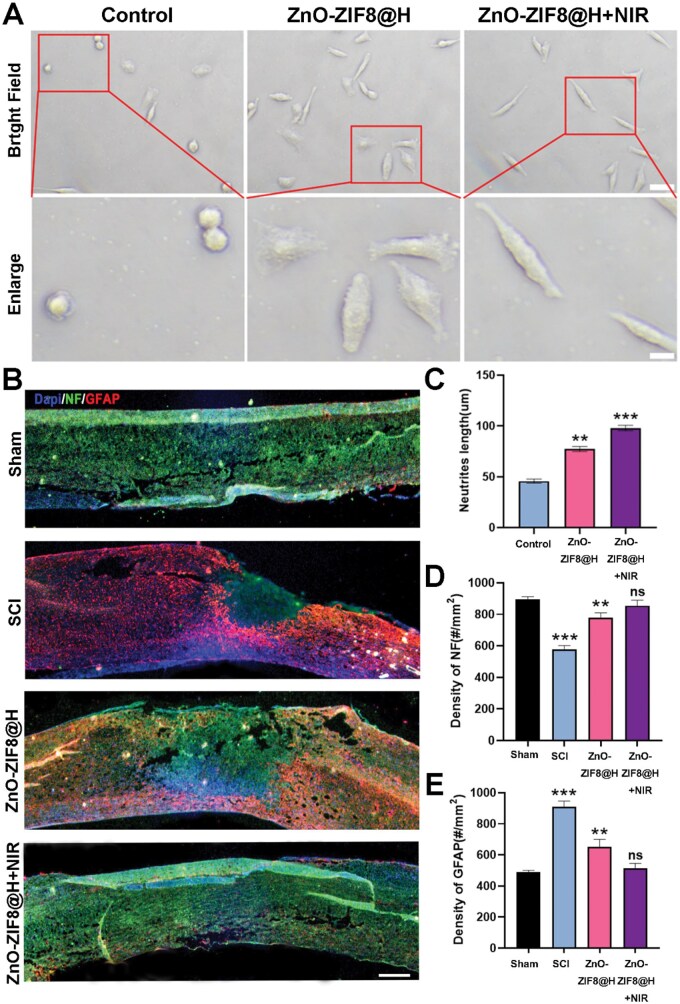
(**A**) The morphological characteristics of PC12 cells observed using a light microscope following treatment with H_2_O_2_, or ZnO-ZIF8@H, or ZnO-ZIF8@H with NIR, bar = 50 μm. (**B**) Immunofluorescence images of spinal cord tissue with neurofilaments (NF) labelled in green, alongside astrocytes glial fibrillary acidic protein (GFAP) labelled in red, bar = 500 μm. (**C**) To evaluate the axon length, the cell branches are counted. (**D**, **E**) Quantitative analysis of NF and GFAP. (*n* = 3, *P < 0.05, **P < 0.01, ***P < 0.001).

In the control group, the cells showed typical stress-induced morphological alterations, characterized by a reduction in cell perimeter and a tendency toward rounded shapes. In the ZnO-ZIF8@H group, cells gradually exhibited a polarized elongated phenotype, with an increased aspect ratio. In the ZnO-ZIF8@H + NIR group, the morphological parameters of the cells were restored, with axonal growth and a network-like extension observed. *In vitro*, ZnO-ZIF8@H combined with NIR irradiation enhanced axonal growth in PC12-simulated neuronal cells (Figure 8A and C). *In vivo*, the use of ZnO-ZIF8@H + NIR significantly promoted the growth of neurons marked by NF, while also notably inhibiting glial scar formation, indicated by GFAP markers (Figure 8B, D and E). Overall, ZnO-ZIF8@H effectively inhibited the ferroptosis process in cells after spinal cord injury. As ferroptosis was suppressed, the oxidative stress response within the cells was alleviated, and the apoptosis process was also inhibited. Ultimately, this effectively promoted the regeneration of nerve cells and suppressed glial scar formation.

To further investigate the inflammatory environment and tissue structural integrity of spinal cord injury sites, Hematoxylin and eosin (H&E) staining was performed on spinal cord injury areas treated with different treatments. As shown in Supplementary Figure S11, the SCI group had not yet formed a complete organizational structure. Although the ZnO-ZIF8@H group has a certain structural framework, there is a significant infiltration of inflammatory cells. In contrast, the ZnO-ZIF8@H + NIR group showed a tightly and regularly arranged tissue structure, similar to the Sham group in terms of organizational integrity. This is consistent with the results of spinal cord injury repair *in vivo*, indicating that the ZnO-ZIF8@H + NIR group has significant advantages in promoting tissue repair and structural reconstruction of spinal cord injury. To further assess the effect of spinal cord injury repair from the perspective of neuronal survival, we performed Nissl staining of the spinal cord injury region in different treatment groups. As shown in Supplementary Figure S12, in the SCI group, there was a significant decrease in the number of Nissl bodies in the spinal cord injury area, and the structure of neuronal cytosol was blurred, suggesting severe neuronal loss; in the ZnO-ZIF8@H group, some of the Nissl bodies were retained, but the density of neurons was still significantly lower than that in the Sham group; in the ZnO-ZIF8@H+NIR group, the distribution of Nissl bodies was uniform and the coloring was clear, and the neuronal cytosol was intact, with a large number of surviving neurons at the edge of the injury area. The ZnO-ZIF8@H + NIR group showed a significant difference, with uniform distribution and clear coloration of Nissl bodies, complete morphology of neuronal cytosol, and a large number of surviving neurons with neuronal density close to that of Sham group. This result is corroborated by the conclusion of the structural integrity of the tissue shown by H&E staining (Supplementary Figure S11): ZnO-ZIF8@H + NIR treatment not only promotes the reconstruction of the spinal cord structure, but also effectively protects neuronal survival and maintains the integrity of the neuronal vesicles, which provides the basis for the functional repair of the spinal cord after the injury.

### ZnO-ZIF8@H enhances motor function recovery in mice after SCI

**Figure 9.**
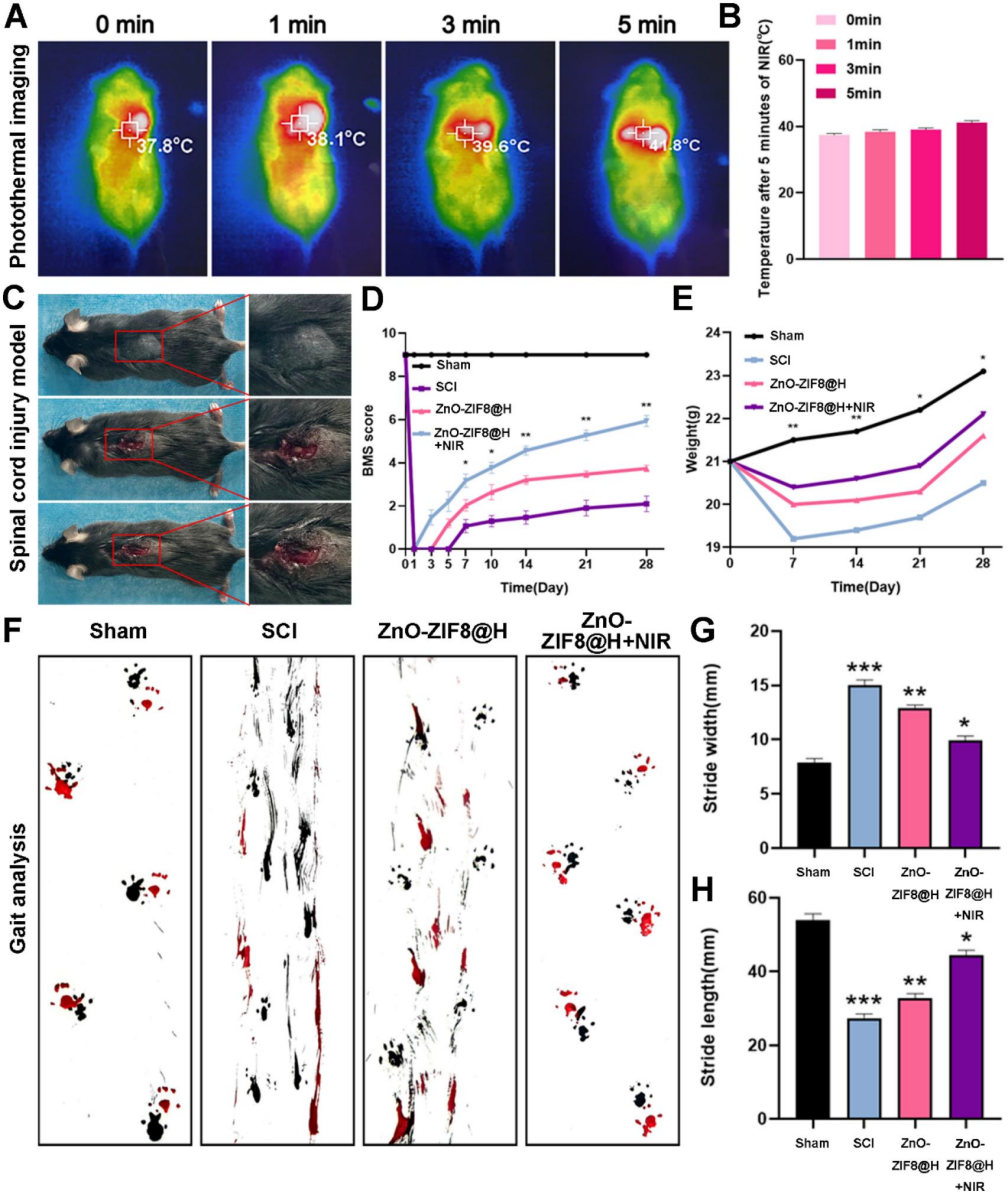
(**A**) Photothermal images of mice after treatments. (**B**) Temperature analysis of the results in (A). (**C**) Image of SCI model mice. (**D**) BMS scores’ analysis. (**E**) Body weight statistics of mice in various groups. (**F**) Representative images of footprint behavior measurements. (**G**, **H**) Quantitative analysis of stride width and stride length in footprint experiments. (*n* = 3, *P < 0.05, **P < 0.01, ***P < 0.001).

In the *in vivo* imaging tests, we observed that the ZnO-ZIF8@H exhibited a superior photothermal effect in mice after being irradiated with near-infrared light, which was not only significant but also had an important impact on the subsequent experimental results (Figure 9A and B). BMS score is an important indicator for evaluating the recovery of motor function in mice with SCI. With scores ranging from 0 to 9, higher score indicates better recovery [41–44]. Further evaluated by the BMS scoring system, the results showed that ZnO-ZIF8@H combined with NIR light treatment significantly assisted the recovery of motor function in mice with SCI, as well as contributing to the recovery of their body weight. Figure 9C–E detail this positive change, and the data show a clear advantage for the treatment group. To more thoroughly verify the restoration of motor function, we also conducted a gait pattern assessment. The results of the analyses clearly demonstrated that the mice treated with ZnO-ZIF8@H + NIR showed a significant improvement in motor function. Our finding further confirms the effectiveness and prospective capability application value of ZnO-ZIF8@H in promoting the recovery of spinal cord injury under NIR light irradiation (Figure 9F–H). H&E staining of the main organs were used for evaluating the biocompatibility of materials. From the perspective of organizational morphology, cells under H&E staining exhibit normal morphology, orderly arrangement and complete structure. In terms of inflammatory response, there is less infiltration of inflammatory cells. When evaluating the functional status of cells, H&E staining shows normal organelles [45–49]. Additionally, H&E staining was performed on cardiac, hepatic, splenic, pulmonary and renal tissues from murine specimens. In each treatment group, no abnormal tissue changes were observed. This suggests that ZnO-ZIF8@H has good *in vivo* biocompatibility and does not exhibit significant tissue toxicity (Supplementary Figure S13). Finally, we injected the mixed hydrogel into the dorsal side of rats via subcutaneous injection (Supplementary Figure S14). The experimental results showed that the hydrogel content gradually decreased due to metabolic action in the body. During this process, no swelling or necrosis was observed in the tissue surrounding the hydrogel. In summary, this study consistently demonstrated, by multiple assessment means, that ZnO-ZIF8@H under near-infrared irradiation is effective in facilitating the restoration of locomotor capabilities in mice with SCI, providing new possibilities for future therapeutic strategies.

## Conclusion

Photothermal responsive antioxidant hydrogels have become a research hotspot because of their broad application prospects in biomedical fields. Based on the existing studies on functional antioxidant hydrogels, this study innovatively proposed a ZnO-ZIF8 nanozymes functionalization strategy and successfully constructed a hydrogel platform with both NIR responsiveness and ROS scavenging ability for SCI treatment. Specifically, we functionalized the injectable PVA-Alg hydrogel (ZnO-ZIF8@H) with ZnO-modified MOFs (ZnO-ZIF8), which significantly enhanced its comprehensive performance. The characterization results show that the functionalized hydrogels have good porous structure and excellent mechanical properties. In addition, under NIR laser irradiation, ZnO-ZIF8@H exhibited high photothermal conversion and significant ROS removal efficiency, which provided a new idea for antioxidant photothermal synergistic treatment of SCI. Through *in vitro* and *in vivo* experiments, it was demonstrated that ZnO-ZIF8@H significantly reduced oxidative stress and apoptosis in damaged neurons. By modulating the ROS microenvironment, it inhibited ferroptosis, which was verified by detecting markers such as GPX4 and HO-1. In addition, measurements of MDA, SOD and GSH levels in spinal cord tissues showed that ZnO-ZIF8@H was effective in mitigating spinal cord injury induced oxidative stress at the molecular level under NIR radiation. The efficacy of the nano-systems in inhibiting neuronal apoptosis was further confirmed by TUNEL staining and Cleaved-Caspase3 immunofluorescence. The unique nanostructure and photothermal conversion ability of ZnO-ZIF8@H were superior under NIR radiation. ZnO-ZIF8@H controlled the release of Zn^2+^, and these Zn^2+^ play key roles in alleviating ferroptosis, mitigating oxidative stress, inhibiting neuronal apoptosis, as well as promoting nerve regeneration. In the SCI mouse model, this resulted in reduced scarring at the injury site and enhanced recovery of motor function. Overall, loading ZnO@MOFs nanozymes into PVA-Alg injectable hydrogels resulted in good photothermal antioxidant properties and improved hydrogel functionality. The strategy of ZnO@MOFs nanozymes to enhance the performance of the injectable hydrogels provides an important idea and technical reference for the design of novel light-responsive antioxidant functional platforms.

## Supplementary Material

rbaf095_Supplementary_Data
