# Supplementary material for: Promoting spinal cord injury repair by using ZnO@MOFs nanozymes functionalized hydrogel through the ROS microenvironment regulating pathway
Source: Regen Biomater. 2025 Sep 13;12:rbaf095. doi: 10.1093/rb/rbaf095 (PMC12582391; doi:10.1093/rb/rbaf095)
Supplement: rbaf095_Supplementary_Data [file rbaf095_supplementary_data.docx]

**Supporting Information**


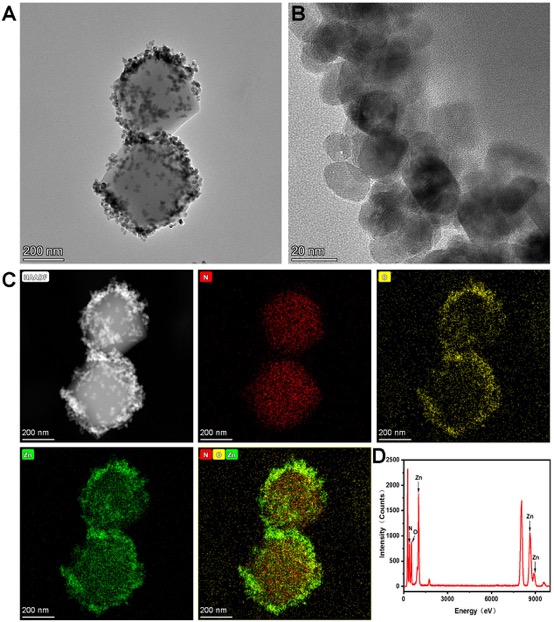


**Figure S1.** (A-B) TEM image of ZnO-ZIF8. (C) High-angle annular dark-field image and TEM element mapping images of ZnO-ZIF8. (D) EDS spectrum of ZnO-ZIF8.


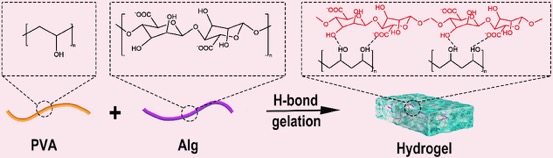


**Figure S2.** Schematic illustration of the formation of PVA-Alg hydrogel by H-bond networks.


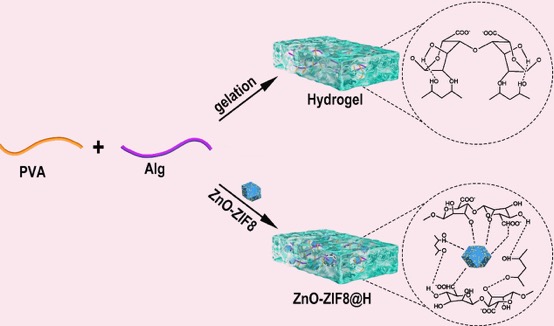


**Figure S3.** Schematic illustration of intermolecular force in the gel networks of pure hydrogel and ZnO-ZIF8@H.


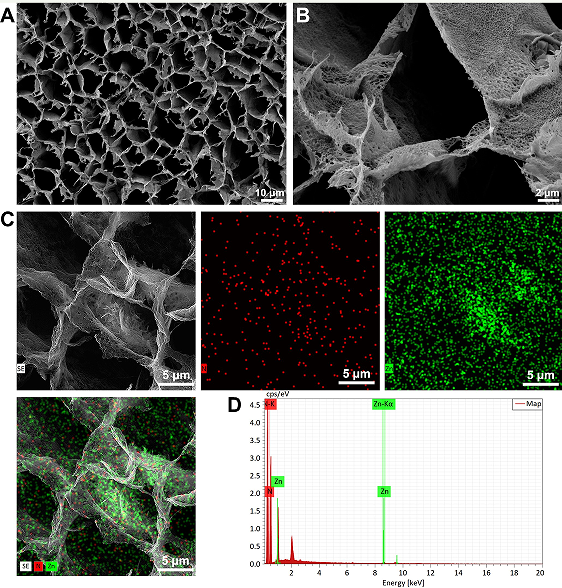


**Figure S4**. (A)SEM image of the ZnO-ZIF8@H, scale bar is 10 μm. (B) Magnified SEM image of the ZnO-ZIF8@H, scale bar is 2 μm. (C) SEM image and element mapping images of ZnO-ZIF8@H, scale bars are all 5 μm. (D) EDS analysis of the ZnO-ZIF8@H.


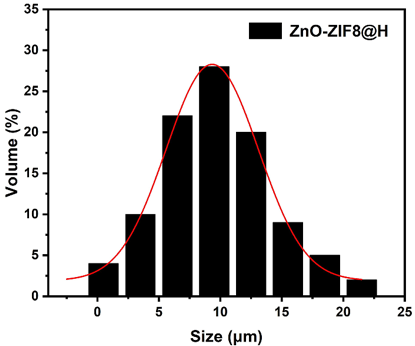


**Figure S5.** Pore size distribution of ZnO-ZIF8@H.

To measure the photothermal conversion efficiency, ZnO-ZIF8@H was exposed to 808 nm radiation at 2.5 W/cm^2^ for 5 min, and then the solution was cooled to room temperature. During this process, the temperature of the solution was recorded every 30 s. According to previous reports [1-2], we calculated the photothermal conversion efficiency of the composite hydrogel according to the following formula:

$$\eta=\frac{hS\left( \Delta T_{max,mix}-\Delta T_{\max,H_{2}O} \right)}{I\left( 1-{10}^{-A_{808}} \right)}$$

(1)

$$hS=\sum mC_{p}/\tau_{s}$$

(2)

$$\tau_{s}=-t/ln\theta$$

(3)

$$\theta=\frac{T-T_{surr}}{T_{max}-T_{surr}}$$

(4)

where $h$ is the heat transfer coefficient, $S$ is the surface area of the container. $\tau_{s}$ is the sample system time constant. It should be noted that the mass ratio of polyvinyl alcohol in the composite hydrogel is only 8% and the water is completely encapsulated in the resultant hydrogel, so the specific heat capacity of the hydrogel can be close to that of pure water. In general, in the components of a hydrogel, the mass proportion of water is extremely high. Since the specific heat capacity of water itself is relatively large and much higher than that of other components in the hydrogel, the total heat of the water component in the gel is higher than that of all other components. Therefore, we consider only the mass and specific heat of water. $\Delta T_{max,mix}$and$\Delta T_{\max,H_{2}O}$are the maximum temperature change of composite hydrogel and water, respectively ($\Delta T_{max,mix}=15.2 ℃ ,\Delta T_{\max,H_{2}O}=1.9 ℃$). When the spot area is 1cm^2^, *I* is 2.5 W. A_808_ represents the absorbance of ZnO-ZIF8@H at 808 nm in the aqueous solution (A_808_ = 0.912), *m* is the mass of water (m = 1.5 g), C*_p_* is specific heat capacity of Methanol (C$H_{2}O$= 4.2 J/(g**∙**℃)), and the value of $\tau_{s}$ is 81.94s obtained from Fig. S6. Which means *T*-*T*_surr_ (*T* and *T*_surr_ are the solution temperature and the ambient temperature, respectively). $T_{max}$ is the highest steady state temperature. According to formula (2), we can get hS equals 0.0769. Substituting it into formula (1), we can calculate that η=46.62% for ZnO-ZIF8@H.


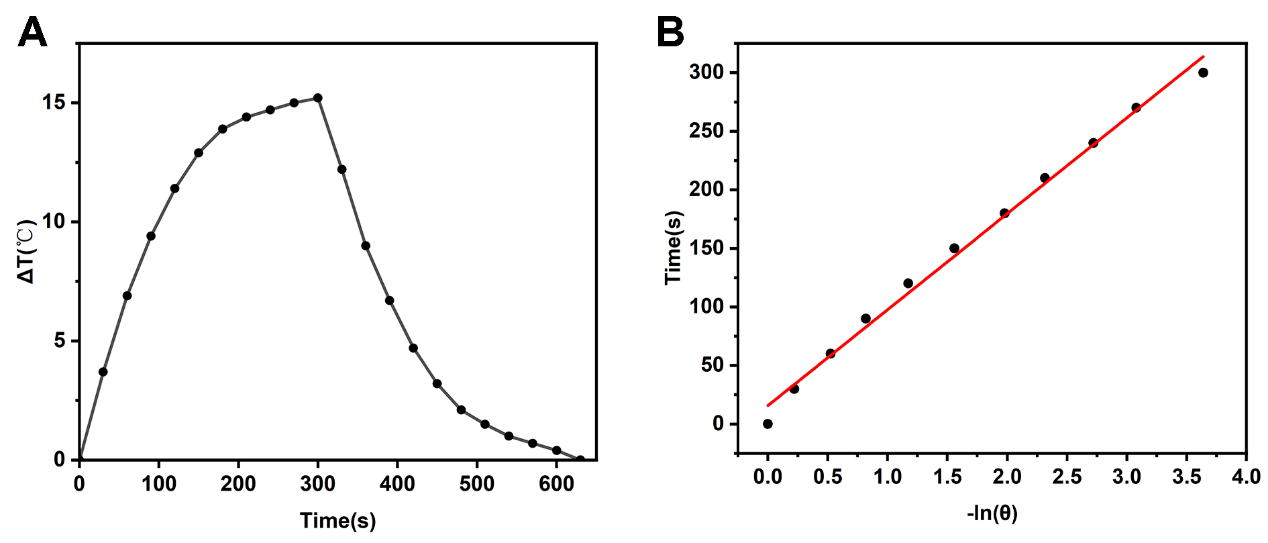


**Figure S6.** (A) The photothermal response of ZnO-ZIF8@H with laser irradiation (808 nm, 2.5 W/cm^2^, 5 min) and then the laser was shut off. (B) Linear time data versus -ln (θ) obtained from the cooling period of NIR laser off.


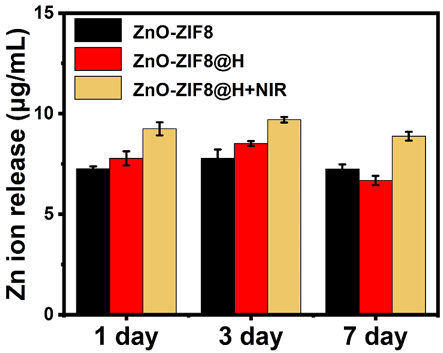


**Figure S7.** Zn ion content released on days 1, 3, and 7 in DMEM.


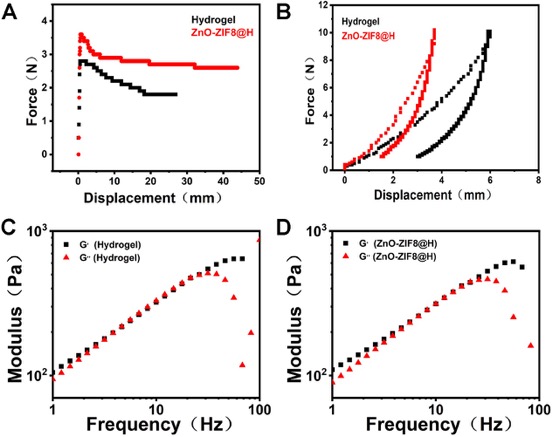


**Figure S8.** (A) Tensile testing of hydrogel and ZnO-ZIF8@H. (B) Compression test of hydrogel and ZnO-ZIF8@H. (C-D) Rheological tests of hydrogel and ZnO-ZIF8@H.


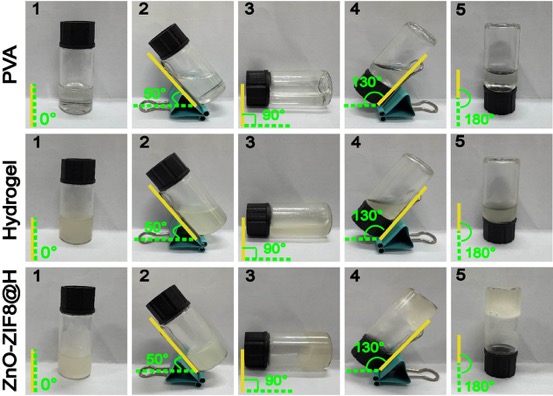


**Figure S9.** Rheological performance images of different groups: PVA solution, PVA-Alg hydrogel, and ZnO-ZIF8@H.


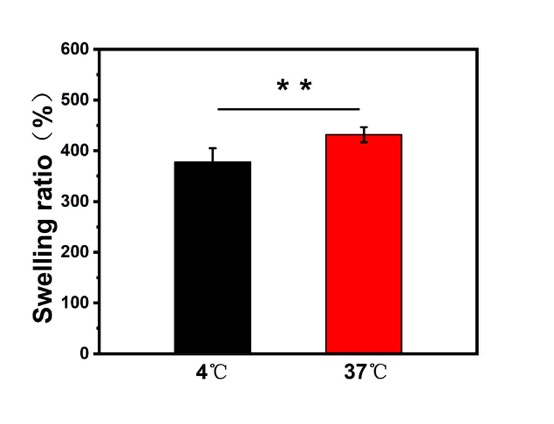


**Figure S10.** The swelling ratio of hydrogel.


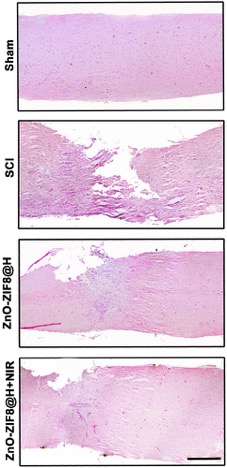


**Figure S11.** Histological analysis of spinal cords across various groups, H&E staining, scale bar = 500 μm.


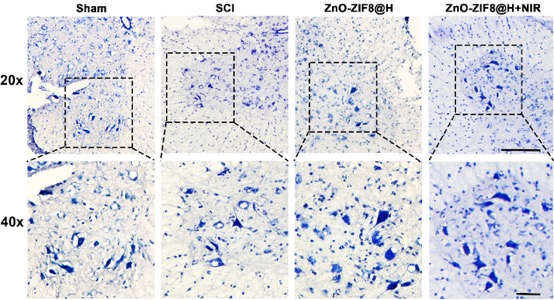


**Figure S12.** After 28 days of treatment with SCI mice, Nissl staining was performed on the ventral horn motor neurons, scale bar (in 20x row) = 100μm; scale bar (in 20x row) = 50μm.


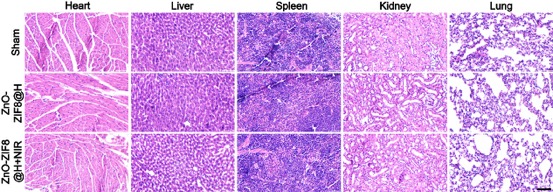


**Figure S13.** Histological sections of H&E staining of the heart, liver, spleen, kidney, and lung with different treatments, scale bar=100 μm.


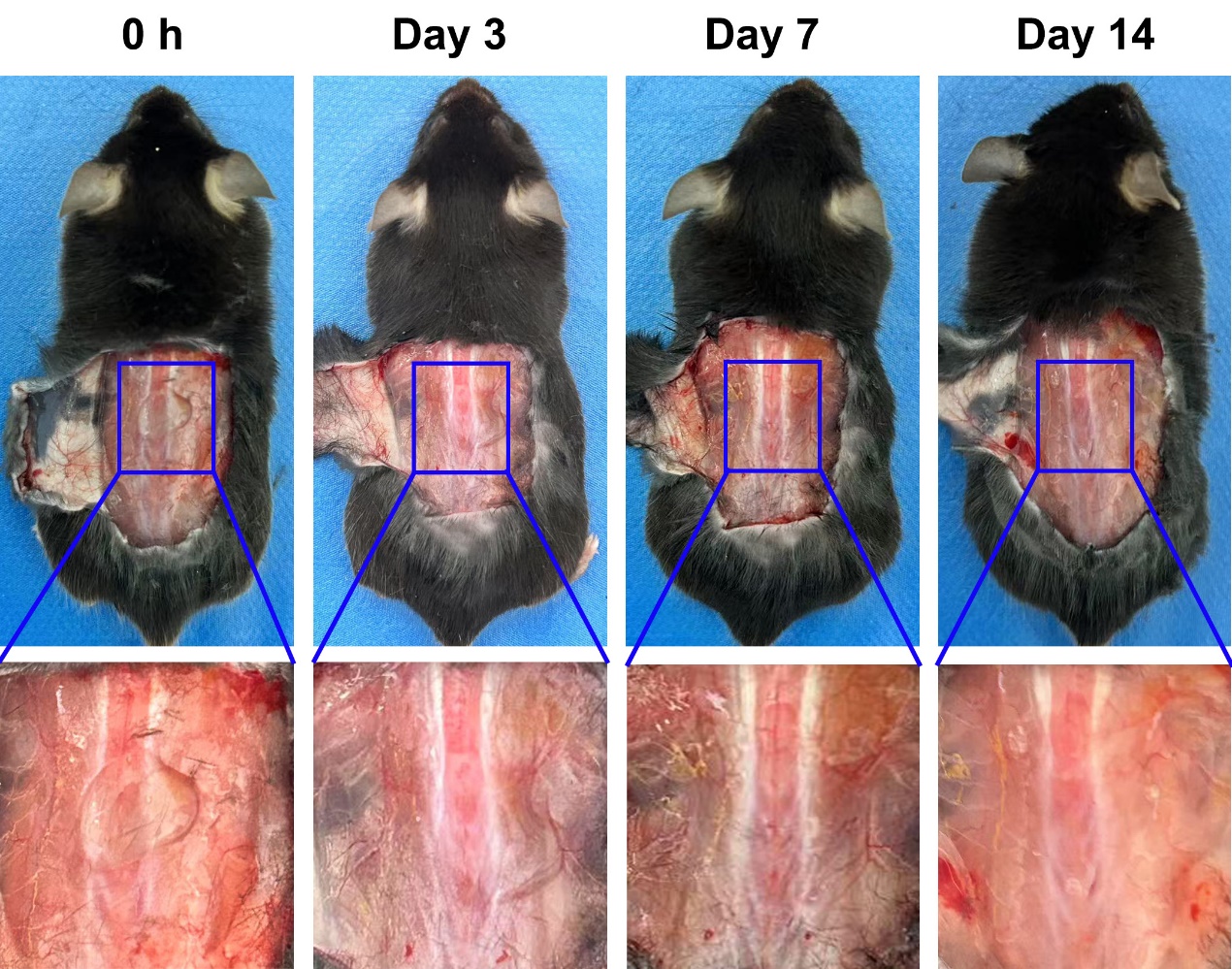


**Figure S14.** Changes in the degradation of composite hydrogels *in vivo* over time.

**References**

1 Chang, L.; Huang, S.; Zhao, X.; Hu, Y.; Ren, X.; Mei, X.; Chen, Z. Preparation of ROS active and photothermal responsive hydroxyapatite nanoplatforms for anticancer therapy. Materials Science and Engineering **2021**; 125: 112098.

2 Huang, Z.; Wang, Y.; Yang, Y.; Gao, Y.; Luo, Y.; Li, X.; Li, N.; Liu, Y.; Zhao, Q.; Li, S.; Zhang, X.-H. Stabilizing Organic Radical Anion in Water by Metal–Organic Frameworks with Enhanced Stability for NIR Photothermal Antibacterial Therapy. ACS Materials Letters **2024**; 6: 535-542.

3. Xiang, E.; Vaquette, C.; Liu, S.; Raveendran, N.; Schulz, B. L.; Nowwarote, N.; Dargusch, M.; Abdal‐hay, A.; Fournier, B. P. J.; Ivanovski, S. Biomimetic Surface Nanoengineering of Biodegradable Zn‐Based Orthopedic Implants for Enhanced Biocompatibility and Immunomodulation. Advanced Functional Materials **2024**; 34: 2410033.

4. Zhu, D.; Cockerill, I.; Su, Y.; Zhang, Z.; Fu, J.; Lee, K.-W.; Ma, J.; Okpokwasili, C.; Tang, L.; Zheng, Y.; et al. Mechanical Strength, Biodegradation, and in Vitro and in Vivo Biocompatibility of Zn Biomaterials. ACS Applied Materials & Interfaces **2019**; 11: 6809-6819.
